# Supplementary material for: The Systems Biology Research Tool: evolvable open-source software
Source: BMC Syst Biol. 2008 Jun 29;2:55. doi: 10.1186/1752-0509-2-55 (PMC2446383; doi:10.1186/1752-0509-2-55)
Supplement: Additional file 1 — SBRT Archive. An archive of the current version of the Systems Biology Research Tool. [file 1752-0509-2-55-S1.zip › sbrt-1.4.0/doc/developers_guide/api/sbrt/shell/text/Format.html]

Format


|  |  |  |
| --- | --- | --- |
| **Overview** \| **Package** \| **Class** \| **Use** \| **Tree** \| **Deprecated** \| **Index** \| **Help** | |  |
| **PREV CLASS**   **NEXT CLASS** | **FRAMES**    **NO FRAMES**     **All Classes** |  |
| SUMMARY: NESTED \| FIELD \| CONSTR \| METHOD | DETAIL: FIELD \| CONSTR \| METHOD |  |


---


## sbrt.shell.text Interface Format

**All Known Subinterfaces:**: AppInputFileLineFormat, BooleanFormat, CatalystFormat, CatalystListFormat, CatalystSetFormat, ChemSpeciesFormat, CoeffFormat, CollectionFormat<E>, ConstraintsFileLineFormat<E>, DirEdgeFormat<N>, DoubleFormat, FbaOptFileLineFormat, FluxCapFormat, FluxomeSolutionFileLineFormat, FluxVectorFormat, IndexPairFormat<R,C>, IntegerFormat, IntervalFormat, LinearCombFormat<V>, LinearCombTermFormat<V>, LongFormat, MapEntryFormat<K,V>, MapFormat<K,V>, MathExprFormat<E,V>, MatrixElementFormat<R,C,E>, PercentFormat, RxnNameExprFormat<E,S>, RxnNameFormat<T>, RxnNameListFormat, RxnNameOrExprFormat, RxnNameSetFormat, SimpleFormat<T>

**All Known Implementing Classes:**: AbstractMapEntryFormat, AbstractMapFormat, AppInputFileLineFormatVersion1, BoolRelFormatV1, BoundedIntervalFormatV1, CatalystFormatV1, CatalystFormatV2, CatalystListFormatV1, CatalystSetFormatV1, CatalystVerifier, ChemSpeciesFormatV1, ChemSpeciesVerifier, CoeffFormatV1, CoeffFormatV2, ColonMapEntryFormatV1, ColonMapFormatV1, ConstraintsFileLineFormatV1, ConstraintsFileLineFormatV2, DirEdgeFormatV1, DoubleFormatV1, DoubleFormatV2, EqualsMapEntryFormatV1, EqualsMapFormatV1, FbaOptFileLineFormatV1, FileFormatFormat, FluxCapFormatV1, FluxomeSolFileLineFormatV1, FluxVectorFormatV1, IndexPairFormatV1, IntegerFormatV1, IntervalFormatV1, IrfFormatV1, IrrevRxnFormatV1, IrrevRxnFormatV1, IrrevRxnFormatV2, LinCombTermFormatV1, LinCombTermFormatV2, LinearCombFormatV1, LinearCombFormatV2, LongFormatV1, MatrixElementFormatV1, NonEmptyStringFormat, PercentFormatVersion1, RevRxnFormatV1, RevRxnFormatV2, RxnNameExprFormatV1, RxnNameFormatV1, RxnNameListFormatV1, RxnNameOrExprFormatV1, RxnNameSetFormatV1, RxnNameVerifier, SenseFormat, SimpleListFormat, SimpleSetFormat, StringFormat, TrueFalseFormat, YesNoFormat

---

``` public interface Format ```

This interface is used to represent formats.
A format provides a way of converting formatted
strings to objects and vice-versa. Since the details of this
process could vary a great deal, no methods have been
specified in this interface. This ensures maximum flexibility,
while still providing a unifying framework for classes
with this type of functionality.

**Author:**
:   This interface was written and documented by
    Jeremiah Wright while in the Wagner lab.

---

---


|  |  |  |
| --- | --- | --- |
| **Overview** \| **Package** \| **Class** \| **Use** \| **Tree** \| **Deprecated** \| **Index** \| **Help** | |  |
| **PREV CLASS**   **NEXT CLASS** | **FRAMES**    **NO FRAMES**     **All Classes** |  |
| SUMMARY: NESTED \| FIELD \| CONSTR \| METHOD | DETAIL: FIELD \| CONSTR \| METHOD |  |


---
